# Burnout, emotional distress and sleep quality among Chinese psychiatric healthcare workers during the COVID-19 pandemic: a follow-up study

**DOI:** 10.3389/fpubh.2023.1272074

**Published:** 2023-12-21

**Authors:** Na-na Xiong, Teng-teng Fan, Qi Liu, Kurt Fritzsche, Rainer Leonhart, Barbara Stein, Christiane Waller, Markus M. Müller

**Affiliations:** ^1^NHC Key Laboratory of Mental Health (Peking University), National Clinical Research Centre for Mental Disorders (Peking University Sixth Hospital), Peking University Sixth Hospital, Peking University Institute of Mental Health, Peking University, Beijing, China; ^2^Department of Psychosomatic Medicine and Psychotherapy, Faculty of Medicine, Center for Mental Health, Medical Centre - University of Freiburg, Freiburg, Germany; ^3^Department for Social Psychology and Methodology, Institute of Psychology, University of Freiburg, Freiburg, Germany; ^4^Department of Psychosomatic Medicine and Psychotherapy, Nuremberg General Hospital, Paracelsus Medical University, Nuremberg, Germany; ^5^Social and Organizational Psychology, Catholic University of Eichstätt-Ingolstadt, Eichstätt, Germany

**Keywords:** anxiety, burnout, depression, healthcare workers, sleep quality, workplace factor

## Abstract

**Background:**

Different from the very early stages of the COVID-19 pandemic, burnout and chronic mental health problems among health care workers (HCWs) has become a challenge. Research is lacking on the relationship between burnout, stress, emotional distress and sleep quality.

**Methods:**

The Chinese center has been involved in the Cope-Corona project since the second survey (T2). Named after the project, a total of three cross-sectional surveys were distributed: T2 (February 16–20, 2021), T3 (May 10–14, 2022), and T4 (December 20–24, 2022). Burnout, depression, anxiety, sleep quality, workplace factors and individual resources were measured. Using the T4 data, we conducted structural equation model (SEM) to examine the mediating role of burnout in predicting emotional distress and sleep quality.

**Results:**

96, 124, and 270 HCWs were enrolled at T2, T3, and T4, respectively. In line with the epidemic trends, the level of perceived COVID-19 related risks was significantly higher at T4, while the feeling of health and safety decreased significantly. At T4, the percentages of participants with clinically significant levels of depression and anxiety symptoms were 18.9% (51/270) and 9.3% (25/270), respectively, while 30.4% (82/270) of them reported poor or very poor sleep quality. According to the SEM, individual resources and workplace factors mainly had an indirect effect in predicting depression and anxiety via burnout. However, neither burnout nor stress was a mediator or predictor of sleep quality. Instead, individual resources, positive workplace factors, and younger age had a direct effect in predicting good sleep quality.

**Conclusion:**

Measures designed to enhance workplace factors and individual resources should be implemented to improve psychosomatic wellbeing of HCWs.

## 1 Introduction

After the COVID-19 pandemic first broke out in Wuhan, China (1), the Chinese government subsequently implemented strict quarantine and isolation measures to control the spread of the virus afterwards. These measures were successful in containing the outbreak, and by mid-2020 the number of new cases had dropped significantly, with only occasional small outbreaks due to the emergence of new variants (1, 2). Therefore, the first phase of the COVID-19 epidemic trends in China was characterized by a combination of strict government measures and relatively low numbers of new cases compared with other countries around the world (1, 3). However, due to the highly transmissible new variants and the reduced effectiveness of public health measures, an unprecedented outbreak started in November 2022.

The mental health impact of the COVID-19 pandemic on healthcare workers (HCWs) has been well documented in the literature (4). However, different from the very early stages of the pandemic (5), the phenomenon of overwork, burnout, and chronic mental health problems among HCWs has become a challenge that requires significant attention (6, 7).

Burnout is characterized as a psychological syndrome of emotional exhaustion, depersonalization, reduced personal accomplishment, and professional ineffectiveness (8, 9). Recently, the concept of burnout has been included in the 11th revision of the International Classification of Diseases (ICD-11) as an occupational phenomenon that may affect health status. According to ICD-11, burnout is a syndrome resulting from chronic workplace stress that has not been successfully managed (10). Based on a study conducted in Wuhan, China, approximately one-quarter to one-half of the sampled medical staff reported various dimensions of burnout symptoms (11). A high prevalence of professional burnout has also been reported from other international studies (12–15). In addition, our longitudinal Cope-Corona project found an increase in burnout during the pandemic (16). The results also examined the Job-Demands-Resources (JDR) model by showing that burnout level was associated with high job demands and low individual resources (17, 18).

Emotional distress of anxiety and depression, and sleep disturbances were also common among HCWs. According to a meta-analysis of HCWs in China, the prevalence rates of moderate to severe anxiety, depression, and sleep disturbances during the pandemic were 17, 15, and 15%, respectively (19). In addition, previous research has shown that burnout or high levels of stress have a negative impact on individuals’ sleep and wellbeing (20, 21), and that heavy workloads, poor sleep quality or insufficient sleep may in return increase the risk of burnout (22–24). According to the 12-phase model, depression and insomnia also represent the final stages of burnout and exhaustion (25, 26).

However, the causal relationship and potential mediators among stress, burnout, resources, workload, emotional distress and sleep quality have remained elusive and inconsistent in the existing literature. For instance, a study involving frontline HCWs revealed burnout as a potential mediator and social support as a potential moderator in the association between prolonged working hours and depressive symptoms (27). Another cross-sectional study of French nurses indicated that emotional dissonance and workload indirectly influenced emotional exhaustion through their impact on sleep quality (8). Additionally, studies with medical staff indicated that anxiety and resilience might act as mediators in the association between perceived stress and depression following quarantine (28); conversely, a study involving psychiatric nurses suggested that stress did not exert a significant direct effect on depressive symptoms; instead, occupational burnout and sleep quality fully mediated their association (29). Additionally, a study conducted on German adults found that personal resources, particularly positive affect, played a moderating role in the relationship between stress and pre-sleep arousal (21). In summary, the inconsistent findings across these studies may stem from variations in the chosen variables of interest, as well as differences in study subjects. Furthermore, the preponderance of cross-sectional designs in most of these studies contributes to the complexity of drawing conclusive insights.

Therefore, we aimed to investigate the prevalence and changes in burnout, stress, emotional distress, and sleep quality, as well as work-related factors and individual resources among Chinese psychiatric HCWs during the COVID-19 pandemic. Additionally, we sought to identify factors associated with adverse mental health outcomes, and to explore the potential mediating influence of burnout using structural equation modeling (SEM). Our hypothesis was that the levels of burnout, stress, emotional distress, and unsatisfactory sleep would increase significantly following the outbreak in the T4 survey, and that burnout would play a mediating role in predicting emotional distress and sleep quality.

## 2 Materials and methods

### 2.1 Study design and participants

This study served as an important component within the framework of the Cope-Corona project, which was designed to investigate how medical staff have handled the challenged posed by the coronavirus pandemic, and to examine their resources and coping strategies. The overall project was a longitudinal, multi-center, international research established with the support of the European Association of Psychosomatic Medicine (EAPM). As reported in our prior publications (16, 30), the project encompassed three surveys: T1 in 2020, T2 in 2021, and T3 in 2022. Participation in the project extended across hospitals in Ireland, Andorra, Spain, Germany, Italy, Romania, Iran, Poland, and China.

**Figure 1:**
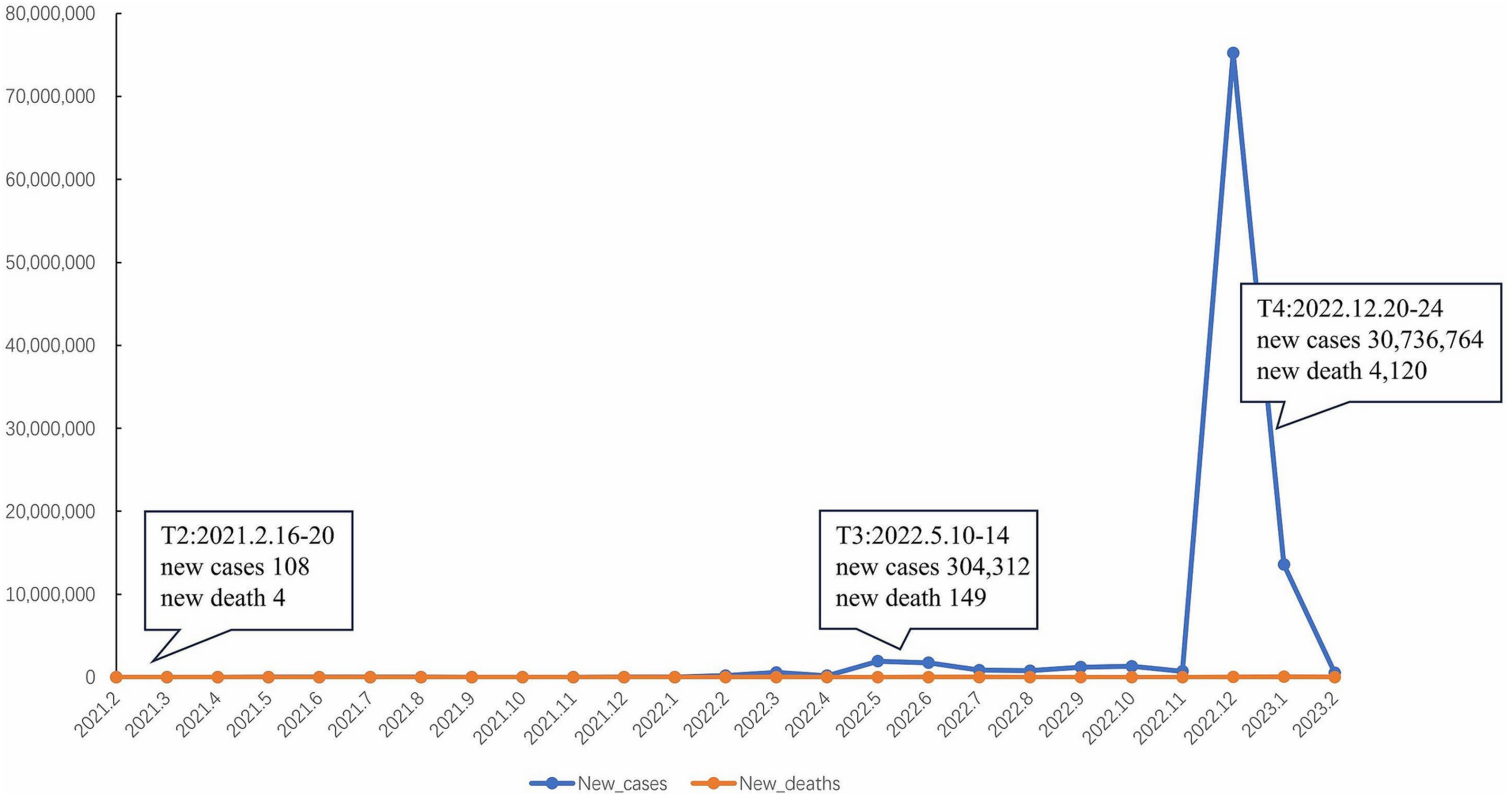
Trends in the number of new confirmed cases of COVID-19 and new deaths in China according to World Health Organization (WHO), and the time points of three surveys.

This study focused only on the follow-up data of medical staff from a Chinese psychiatric hospital in Beijing. We obtained ethical approval from the Peking University Sixth Hospital (No. 2020–65). Prior to the survey, all participants were provide with the study’s introduction to ensure full comprehension, and their consent was obtained by clicking the “I agree” button to proceed with questionnaires. Alternatively, participants had the option to decline participation by clicking the “I disagree” button. Indeed, the Chinese center actively joined the project starting from T2. Nevertheless, as shown in Figure 1, according to the data from World Health Organization (WHO) (1), unlike the trends worldwide, only small outbreaks occurred in China during the first two surveys (T2 February 16–20, 2021 and T3 May 10–14, 2022). Therefore, an additional T4 survey was conducted following the unprecedented outbreak in China in December 2022. Thus, this study focuses on data from three surveys (T2, T3, and T4) in China.

We designed the study as an online survey using the h6world platform^1^ (31). This platform was supported by the Peking University and was used to ensure the safety and privacy of our data. If missing values were detected, the online system would remind participants to complete. As a result, for each variable of interest, no missing value was detected. The QR code was distributed in the staff group via WeChat. Normally, this WeChat group was used to disseminate important announcements in the hospital. All 500 employees of the psychiatric hospital were included in the group, including doctors, nurses, medical-technical staff, administrative workers, psychologists, researchers, trainee and medical students. Therefore, all adult employees of the psychiatric hospital were invited to participate in the survey at three measurement time points, regardless of their previous participation. The survey was anonymized. Subjects were asked to provide a self-generated identification code, which was a combination of the first letter of the participants’ parents’ first names, place of birth and house number, and the last digit of the year of birth, to match subjects at different assessment points.

### 2.2 Instruments

The following constructs were measured using established questionnaires. The validity and reliability of the Chinese versions of questionnaires have been reported in our previous publications (16, 30).

#### 2.2.1 Demographic and occupational variables

Variables of gender, Age, hospital position, work experience, and The previous history and treatment of mental illness were measured.

#### 2.2.2 Workplace factors

1. Contact with COVID-19 patients. Participants were asked the frequency of dealing directly with coronavirus-infected patients or suspected cases in their work. Responses were scaled from 1 = “not at all,” 2 = “rarely,” 3 = “sometimes,” and 4 = “very much.”
2. Risk perception. Personal risk regarding the coronavirus was measured with three items indicating the likelihood of becoming infected (1 = “extremely unlikely” to 5 = “extremely likely”), the danger of being infected oneself (1 = “completely harmless” to 6 = “I have been infected already”), and concern about infecting people in one’s personal life (1 = “very little” to 5 = “very much”). Cronbach’s alpha in the sample was 0.56. We used the average score to indicate the severity of the risks involved.
3. Workload. At T4, three additional items were added to measure the changes in workload during the pandemic. The questions were based on a study in Germany (32). Working conditions were rated on a 5-point scale from 0 “strongly disagree” to 4 “strongly agree”: “I work more than before the COVID-19 pandemic”; “There are sufficient staff for the current work load”; and “I can recover sufficiently during my free time.” The last two questions were scored in reverse order. A total sum score was used to indicate the level of workload, with higher scores indicating a heavier workload. Cronbach’s alpha in the sample was 0.48.
4. Health and safety in the workplace. Two items about the availability of personal protective equipment and the subjective feeling about the confident to stay healthy at work were rated on 5-point scales to measure health and safety in the workplace. A higher mean score indicates stronger feelings of health and safety. Cronbach’s alpha in the sample was 0.67.
5. Support in the workplace. Five items were used to reflect this construct, including the quality of within-team collaboration, cross-team communication, trust in supervisors, recognition from supervisors, and information provided by the hospital. All items used 4-point scales from 1 = “strongly disagree,” 2 = “disagree,” 3 = “agree,” and 4 = “strongly agree.” Cronbach’s alpha in the sample was 0.89.

#### 2.2.3 Individual resources

1. Self-compassion. The State Self-Compassion Scale-Short (SSCS-S) was adopted to reflect the capacity for self-compassion (33, 34). It refers to the ability to hold a kind and understanding attitude toward one’s stressful experiences, and to recognize it as part of the common human experience. Responses were scored on a 5-point scale (1 = “not at all true for me” to 5 = “very true for me”). The mean score was used to indicate the ability to be self-compassionate. Cronbach’s alpha in the sample was 0.67.
2. Sense of coherence. The newly developed 3-item version of the sense of coherence (SOC) scale, the brief assessment of sense of coherence (BASOC), was used to reflect the ability to comprehend, manage and make sense of an experience (35). Responses were scored on a 7-point scale, with higher mean scores indicating better SOC. Cronbach’s alpha in the sample was 0.69.
3. Altruism. A single item was used to measure COVID-19 related altruistic acceptance of risk. The item used a 5-point scale ranging from “fully agree” (5) to “completely disagree” (1). It has been shown to be a relevant factor against burnout regarding SARS viruses (36, 37).

#### 2.2.4 Psychosomatic wellbeing

1. Perceived stress. The Perceived Stress Scale (PSS-4) was used to assess levels of psychological distress over the past month, especially regarding feeling of controllability and confidence in handling stressful situations. The psychometric properties of the PSS-4 are acceptable across cultures and countries (38). Responses for the four items were rated on a 5-point scale. The total score ranged from 0 to 16, with higher scores indicating higher levels of stress. According to our previous study and other similar studies, a cut-off score of 6 was recommended to categorize participants with high levels of stress (30). Cronbach’s alpha in the sample was 0.89.
2. Burnout. We evaluated the level of burnout using the two-item version of the Maslach Burnout Inventory (MBI-2) (39). The two items represented the two dimensions of burnout, emotional exhaustion and depersonalization. Items were rated on a 7-point scale ranging from 0 (“Never”) to 6 (“Every day”). As reported, a cut-off score of 4 was used to represent a high level of burnout (16). Cronbach’s alpha in the sample was 0.75.
3. Depression and anxiety. Depression and anxiety were measured using the 4-item brief health questionnaire, which consists of a 2-item depression scale (PHQ-2) and a 2-item anxiety scale (GAD-2) (40, 41). For each scale, the total sum scores range from 0 to 6. A cut-off score of 3 has been suggested to detect probable cases of clinically significant levels of depressive and anxiety symptoms. Cronbach’s alpha in the sample was 0.88.
4. Sleep quality. A single item from the Pittsburgh Sleep Quality Index (PSQI) (42) was used to assess overall sleep quality over the previous 2 weeks. It was rated on a 4-point scale (0 = “very good,” 1 = “fairly good,” 2 = “fairly bad,” 3 = “very bad”). This item was newly added at T4.

### 2.3 Statistical methods

To compare demographic and occupational features between the three groups, the χ^2^-test was used for categorical variables. To control for the potential confounding effects of position and work experience, analysis of covariance was used to compare workplace factors, individual resources and psychosomatic wellbeing between the three time points. The Bonferroni correction was adopted for multiple comparisons, although it has been criticized sometimes, particularly in cases of small sample sizes or a high number of conducted tests. In our study, we maintained a fair sample size, and the number of tests conducted was moderate. Therefore, the application of the Bonferroni correction is not expected to significantly impact the magnitude of effect sizes (43, 44). A *p* < 0.05 (two-tailed) was considered significant.

To test the mediating role of burnout, multiple linear regressions were first performed on the T4 sample to find potentially significant predictors for the mediators, and then predictors for the outcomes from both the latent variables and the mediators. We adopted the stepwise method, with a *p* < 0.05 required to enter and less than 0.10 required to remain in the model. Second, path models were constructed and tested within the T4 sample using the bootstrapped maximum likelihood method. Pathways with a non-significant contribution (*p* > 0.05) were deleted. Latent variables with a loading less than 0.4 were also removed. Reasonable correlations were allowed according to the modification indices to further improve the model fit. Model fit was assessed using Chi-square difference tests and inspection of the Root Mean Square Error of Approximation (RMSEA) and the Comparative Fit Index (CFI). A value of 0.05 or less for RMSEA was considered to be very good, while 0.05–0.08 was acceptable (39). A value of 0.95 or greater for CFI was considered to be adequate (40). Statistical analyses were performed using IBM SPSS Statistics 24.0 and AMOS 23.0 (45).

## 3 Results

### 3.1 Demographic and occupational characteristics

**Figure 2:**
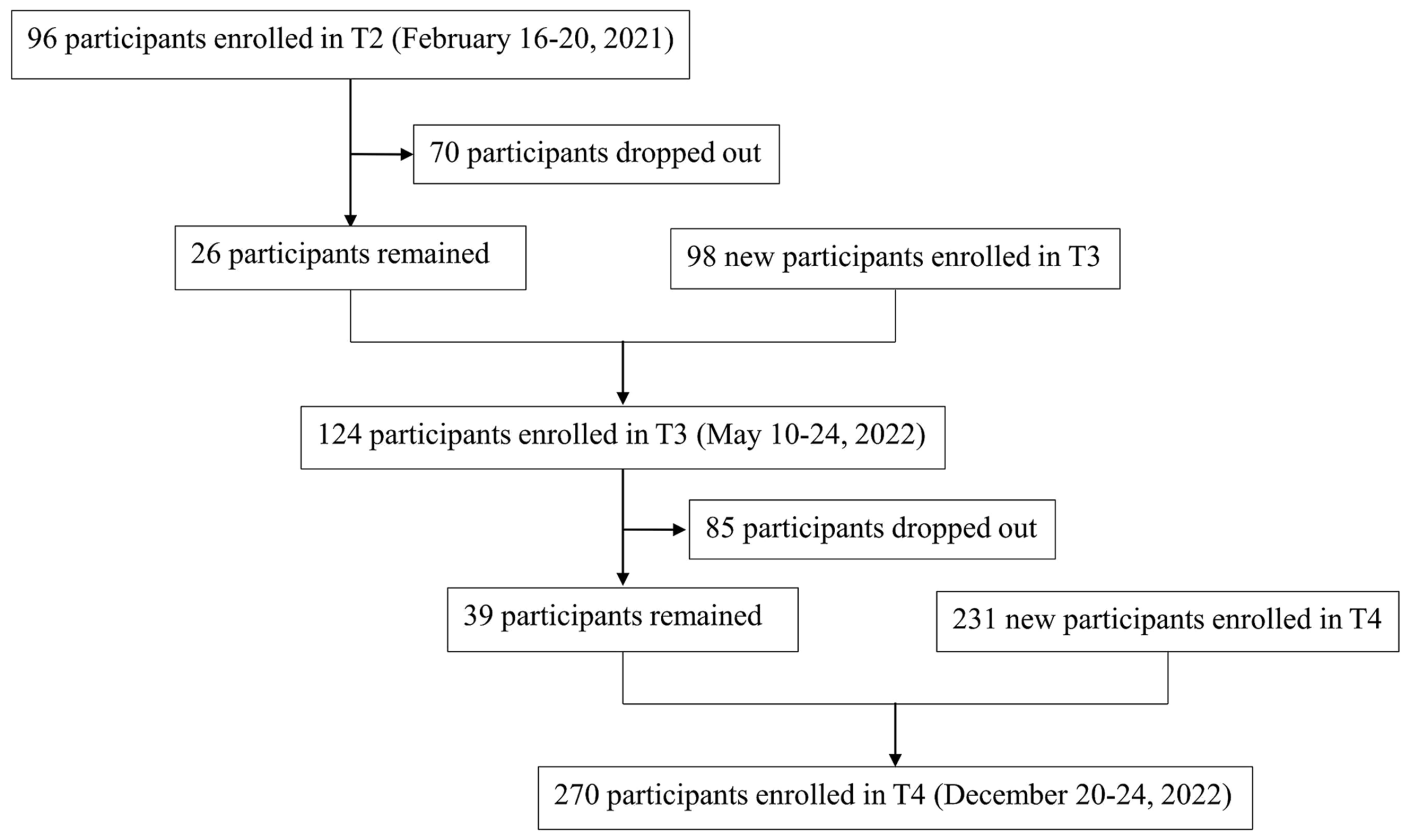
Flow diagram and the time points of three surveys.

As a result (see Figure 2), 96, 124, and 270 HCWs were enrolled at T2, T3, and T4, respectively. Since the total number of HCWs in this hospital was 500, the response rates were estimated to be 19.2, 24.8, and 54.0%, respectively. Unfortunately, according to their self-generated identification code, only less than a third of them completed all three surveys. Therefore, the longitudinal cohort was underrepresented, and data from the three cross-sectional surveys were analyzed and compared separately.

As shown in Table 1, the majority of HCWs in our sample were female, middle-aged, nurses and doctors, and HCWs with more than 6 years’ experience. In addition, between 3.2 and 8.5% of HCWs in this psychiatric hospital reported a positive history of mental disorders, and between 2.4 and 6.7% of them had received relevant treatment.

**Table 1:** Demographic and occupational characteristics of HCWs.

Variables	T2 (*n* = 96)	T3 (*n* = 124)	T4 (*n* = 270)	χ^2^	*p*
Female n(%)	75(78.1)	91(73.4)	208(77.0)	0.8	0.657
Age groups n(%)				9.2	0.323
<26 years old	15(15.6)	13(10.5)	42(15.6)		
26–35 years old	50(52.1)	52(41.9)	115(42.6)		
36–45 years old	21(21.9)	33(26.6)	63(23.3)		
46–55 years old	9(9.4)	21(16.9)	45(16.7)		
>56 years old	1(1.0)	5(4.0)	5(1.9)		
Position n(%)				19.8	**0.011**
Doctor	41(42.7)	48(38.7)	84(31.1)		
Nurse	18(18.8)^2^	43(34.7)^1^	102(37.8)^1^		
Technician	8(8.3)	13(10.5)	32(11.9)		
Administrator	7(7.3)	7(5.6)	20(7.4)		
Others	22(22.9)^1^	13(10.5)^2^	32(11.9)^2^		
Job experience n(%)				9.9	**0.042**
<3 years	27(28.1)	28(22.6)	80(29.6)		
3–6 years	22(22.9)	14(11.3)	37(13.7)		
>6 years	47(49.0)^2^	82(66.1)^1^	153(56.7)		
Positive history of mental illnesses n(%)	6(6.3)	4(3.2)	23(8.5)	3.8	0.147
Treatment for mental illnesses n(%)	4(5.2)	3(2.4)	18(6.7)	3.4	0.184

Time points of the three surveys: T2 February 16–20, 2021; T3 May 10–24, 2022; T4 December 20–24, 2022. The Bonferroni method was adopted for multiple comparisons: values with ^1^ were significantly higher than values with ^2^, and only values with different superscripts were significantly different from each other. *p*-values in bold type indicate significant differences.

When comparing the three groups, the demographic characteristics were generally comparable across the three surveys, with the exception that the proportion of nurses was higher at T3 and T4, and the proportion of participants with more than 6 years’ experience was higher at T2.

### 3.2 Workplace factors

In line with the epidemic trends in Beijing, the frequency of contact with patients with COVID-19 has increased significantly at T4, with 59.6% of mental health professionals reporting frequent contact with them (see Table 2). In addition, only 2.4% (3/124) of participants reported coronavirus infection at T3, but 83.5% (226/270) of them reported infection at T4.

**Table 2:** Epidemic-related working burden, workplace factors, individual resources and psychosomatic wellbeing of HCWs.

Variables	T2 (*n* = 96)	T3 (*n* = 124)	T4 (*n* = 270)	*F*/χ^2^	*p*
Workplace factors					
Corona contact n(%)				154.6	**<0.001**
Hardly any	94(97.9)	114(91.9)	109(40.4)		
Much	2(2.1)^2^	10(8.1)^2^	161(59.6)^1^		
Risk perception	2.6 ± 0.8^1^	2.7 ± 0.7^1^	4.0 ± 0.6^2^	272.4	**<0.001**
Workload	–	–	5.4 ± 2.5	–	–
Health and safety	2.0 ± 0.6^1^	1.9 ± 0.6^1^	1.6 ± 0.7^2^	18.9	**<0.001**
Workplace support	3.0 ± 0.5	3.0 ± 0.5	3.0 ± 0.5	0.1	0.948
Individual resources					
Self-compassion	3.6 ± 0.6	3.7 ± 0.6	3.7 ± 0.6	0.4	0.649
Sense of coherence	5.2 ± 1.4	5.4 ± 1.2	5.5 ± 1.3	1.0	0.365
Altruism	3.7 ± 0.7	3.7 ± 0.8	3.6 ± 0.9	0.6	0.533
Psychosomatic wellbeing					
Stress	5.8 ± 2.2	6.3 ± 2.4	6.0 ± 2.5	0.9	0.405
Burnout	3.1 ± 2.9	3.2 ± 2.7	2.9 ± 2.8	0.5	0.621
Depression	1.2 ± 1.1^2^	1.4 ± 1.3	1.6 ± 1.3^1^	3.5	**0.030**
Anxiety	1.1 ± 1.3	1.2 ± 1.2	1.2 ± 1.3	0.8	0.468
Sleep quality	–	–	1.2 ± 0.8	–	–

Time points of the three surveys: T2 February 16–20, 2021; T3 May 10–24, 2022; T4 December 20–24, 2022. All comparisons above have been controlled for the covariate of position and job experience, and the Bonferroni method was adopted for multiple comparisons: values with ^1^ were significantly higher than values with ^2^, and only values with different superscripts were significantly different from each other. *P*-values in bold type indicate significant differences.

Concerning workload, at T4 only 25.9% (70/270) of them agreed or strongly agreed that they were able to recover sufficiently in free time, while 18.5% (50/270) of HCWs agreed or strongly agreed that they were working more than before the pandemic.

Compared to T2 and T3, the level of perceived COVID-19 related risks has also increased significantly at T4, while the feeling of health and safety at work decreased significantly at T4. However, the level of perceived support from the workplace remained at similar levels across the three surveys.

### 3.3 Individual resources and psychosomatic wellbeing

As shown in Table 2, after controlling for the potential confounding factors, three dimensions of individual resources, and psychosomatic wellbeing of stress, burnout and anxiety, remained at similar levels across the three surveys. However, it was notable that the depression levels were significantly higher at T4 than at T2.

At T4, the percentages of healthcare workers with a high level of stress and burnout were 58.9% (159/270) and 29.6% (80/270), respectively. In addition, the percentages of participants with clinically significant levels of depression and anxiety symptoms were 18.9% (51/270) and 9.3% (25/270), respectively, while 30.4% (82/270) of all participants reported poor or very poor sleep quality. Moreover, the correlation analyses revealed a significant and moderate association between the level of burnout and both depression (*r* = 0.526, *p* < 0.001) and anxiety (*r* = 0.544, *p* < 0.001).

### 3.4 The mediating role of burnout in predicting psychosomatic symptoms

The mediating role of burnout was tested using data from T4. First, multiple linear regressions were performed to find potentially significant predictors of the mediator (burnout) among latent variables (stress, demographic and occupational characteristics, history of mental illness, workplace factors, and individual resources), and then predictors of the outcomes (psychosomatic symptoms of depression, anxiety and poor sleep quality) from both the latent variables and the mediators.

As a result, we confirmed that variables of stress, working years, workload, support at workplace, and sense of coherence were the independent predictors of burnout, with 37.1% of the total variance explained. In addition, burnout, together with other latent variables, were also the independent predictors of psychosomatic outcomes of depression, anxiety, and sleep quality. However, the predictors of emotional distress of depression and anxiety differed from sleep quality. Therefore, SEMs were constructed separately for the psychosomatic outcomes of emotional distress and sleep quality.

**Figure 3:**
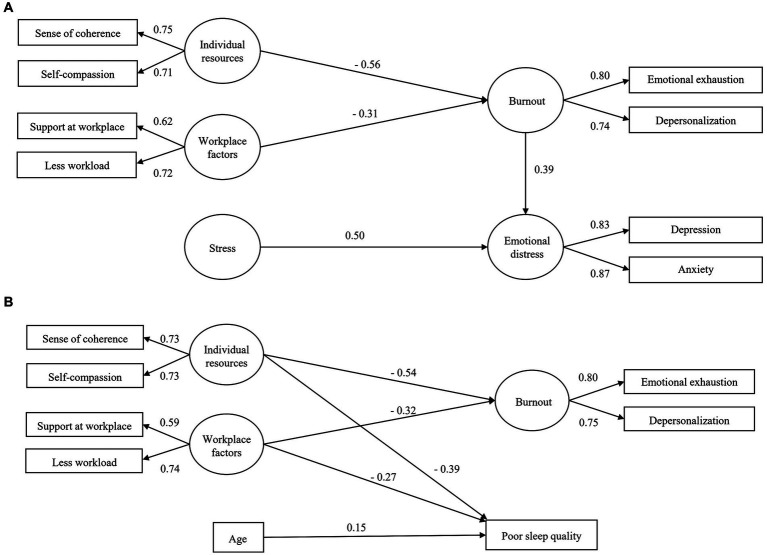
The structural equation model (SEM) of the mediating role of burnout in predicting emotional distress and sleep quality within data from T4 (*n* = 270). **(A)** Burnout mediated the relationship between individual resources, workplace factors and emotional distress, while stress had a direct effect in predicting depression and anxiety. **(B)** Individual resources, workplace factors and age only had a direct effect in predicting sleep quality. Standardized estimates are shown for the significant regression paths. Residual errors are omitted from the figure.

As shown in Figure 3A, individual resources (sense of coherence and self-compassion) and workplace factors (support and workload) mainly had an indirect effect in predicting emotional distress of depression and anxiety via burnout, whereas stress mainly had a direct effect in the association. The loadings of all latent variables were higher than 0.4. The model fit indices were very satisfactory [χ^2^(df) = 62.3 (46), *p* = 0.055, RMSEA (90% CI) = 0.036 (<0.001–0.058), CFI = 0.987].

For sleep quality in Figure 3B, stress was removed from the model due to the non-significant contribution. In addition, no mediating role via burnout was detected. As a result, individual resources and workplace factors only had a direct effect in predicting sleep quality or burnout, separately. Older age also had a direct effect in predicting poor sleep quality. Fit indices of the model were acceptable [χ^2^(df) = 28.7 (16), *p* = 0.026, RMSEA (90% CI) = 0.054 (0.019–0.086), CFI = 0.975].

## 4 Discussion

As part of the Cope-Corona project (16), this follow-up study allowed us to examine trends in the frequency and risks of COVID-19 exposure, workplace factors, and the psychosomatic wellbeing of HCWs before and during the outbreak of the pandemic. We also explored the predictive and mediating factors for emotional distress and sleep quality.

As expected, the frequency of contact with COVID-19 patients, the percentage of infection, the level of perceived risks, and the workload increased significantly during the outbreak of the epidemic compared with the previous period of occasional small outbreaks. However, perceived support from the workplace remained at similarly high levels across the three surveys. Regarding the mental health of HCWs, it is interesting to note that only depression increased significantly during the outbreak period, while levels of stress, burnout, and anxiety were comparable at all three time points.

During the peak of the pandemic, the levels of depression, anxiety, sleep disturbance, and stress were generally comparable to previous international studies (19, 32, 46–48). However, the prevalence of burnout in our study, at 29.6%, was relatively low compared with other studies. For example, 2 years after the pandemic in Wuhan, China, up to 67.09% of medical staff still met the criteria for burnout as defined by the Maslach Burnout Inventory General Survey (49). Studies of HCWs in Ireland and Singapore reported that burnout thresholds, as measured by the Oldenburg Burnout Inventory (OLBI), were reached by 74–80% of respondents (12, 50). However, a number of studies have also reported prevalence similar to our results. For example, a study in Poland reported a prevalence ranging from 27.7% among non-medical staff to 36.5% among nurses (51); 8 months after the initial peak of the pandemic, a US study reported rates of depersonalization and emotional exhaustion of 21.6 and 46.0%, respectively (52). The utilization of diverse scales and cut-off points may potentially contribute to variations in reported burnout prevalences, alongside disparities in healthcare systems, workload conditions, and the severity of the pandemic across distinct research studies. Additionally, another possible reason for the differences in prevalence of burnout should not be neglected: our research focused on a specific group of mental health workers. On the one hand, they faced with unique emotional challenges when caring for patients with mental illness; on the other hand, they were supposed to have better professional skills in managing negative emotions and team communication. Furthermore, the correlation analyses unveiled a significant and moderate association between the degree of burnout and depression as well as anxiety, shedding light on the potential overlap between these constructs. Nevertheless, the observed moderate correlation implies that burnout is more than simply a manifestation of depression or anxiety.

According to the JDR model, high job demands could increase burnout, whereas workplace and individual resources could reduce burnout (17, 18). Therefore, the relatively high level of support at work, and the availability of individual resources in this research may have played an important role in preventing burnout among the psychiatric HCWs during the peak of the epidemic. This phenomenon may be attributed, in part, to the likelihood that mental health workers possess a heightened capacity to mobilize individual resources and facilitate teamwork to cope with challenging experiences. In addition, previous research has suggested that gender may be another factor influencing burnout. For example, a large sample of Chinese nurses showed that women had significantly higher levels of emotional exhaustion, while another study showed that men had significantly higher levels of depersonalization (22.3% vs. 17.9%) (13). However, we did not find a difference in burnout between women and men.

Our study also extends previous research by examining the differential mediating role of burnout in predicting adverse psychosomatic outcomes. According to our results, individual resources and workplace factors influenced depression and anxiety mainly through the mediating effect of burnout, whereas stress level mainly through a direct effect. In contrast to our findings, a small sample study of nurses in Saudi Arabia found that work-related stress had negative indirect effects on emotional wellbeing through compassion fatigue (53). In their study, the concept of compassion fatigue consisted of two dimensions: burnout (exhaustion, frustration and anger) and secondary traumatic stress. Therefore, the different measurements, positions of the medical staff, and the medical system could account for the different mediative role of burnout. Similarly, two other studies of frontline medical staff in Wuhan and Jiangsu, China, both found that long working hours had a negative effect on depressive symptoms via burnout, and that social support was a potential moderator of the association (27, 49). Unfortunately, neither study measured individual resource variables.

Furthermore, although previous literature suggests that burnout may affect sleep quality (25), the relationship among stress, burnout and sleep quality have remained elusive and inconsistent in the existing literature, particularly among HCWs during the COVID-19 (8, 27). According to our results, in contrast to the model of emotional distress, stress and burnout were not significant independent predictors or mediators of sleep quality. Instead, variables related to individual resources, work related factors, and age were able to directly predict sleep quality. Therefore, we hypothesize that, unlike the significant predicting or mediating effect on emotional distress, stress and burnout may not have a direct effect on sleep quality. On the other hand, a study involving German outpatient nurses found that pandemic-related stress predicted a decline in sleep quality and work engagement (54). Similarly, a study conducted in China explored the direct and indirect relationships between perceived stress, anxiety and depression, and sleep quality (55). Their findings revealed that the initial association between perceived stress and sleep quality diminished after introducing anxiety and depression as mediating variables. Moreover, numerous studies have explored factors associated with sleep disturbances among healthcare workers during the pandemic. For example, a meta-analysis suggested that female sex and location in China were associated with fewer sleep disturbances (56), but some other studies also reported that female HCWs slept worse (57). Working long shifts, experiencing a death in the family, and being a frontline worker have also been suggested to be associated with increased odds of insomnia or poor sleep quality (58). In addition, levels of burnout have been found to correlate with insomnia in frontline nurses (59), and burnout has been found to be particularly high in nurses who start to use sleep medication (60).

This study has several limitations. Firstly, only a small proportion of HCWs participated in all three surveys, making it impossible to directly compare changes at an individual level, or to examine the predictive value of certain variables at baseline within the cohort. In fact, the self-generated identification code used was probably one of the causes of the low match rates itself. Because participants’ house numbers may have changed over the last 2 years, and because Chinese participants may think of province, city, or county when filling in information about their place of birth in different surveys. Therefore, the same participant could generate different identification codes in three surveys, leading to a match failure. To overcome this problem, we had to treat the data as three cross-sectional studies, and the regression analyses were only carried out on the T4 sample. Secondly, the workload and sleep quality items were only added at T4, making it impossible to compare across the three surveys. Thirdly, to streamline the survey process and conserve time, concise measurement versions were employed in this study. However, it is acknowledged that this approach may potentially result in an overestimation of outcomes. To address this concern, we have opted for validated versions of the measurements. Fourthly, only HCWs from one psychiatric hospital were recruited, and the results should be interpreted with caution regarding mental health workers from different backgrounds. Compared to HCWs in general hospitals, especially those frontline workers in respiratory, emergency and intensive care units, mental health workers were not directly faced with critically ill or dying patients, but they have to deal with COVID-19 patients with severe mental illness, and at the same time with increased workload and pressure during the outbreak. In addition, as discussed above, psychiatric HCWs were expected to have better professional skills in coping with stress and emotional distress, and to provide psychological support to other colleagues in general hospitals as well as to the general population.

In sum, this study found that during the outbreak, Chinese psychiatric HCWs were under significant pressure, with 58.9 and 29.6% of them reporting high levels of stress and burnout, respectively. In addition, we found that individual resources of self-compassion and sense of coherence, and positive workplace factors of workplace support and lower workload were the independent significant protective factors against emotional distress and poor sleep. However, unlike the significant predicting or mediating effect on emotional distress, stress and burnout may not have a direct effect on sleep quality. Future studies should further explore the associated factors of sleep quality with the longitudinal data.

## Data availability statement

The raw data supporting the conclusions of this article will be made available by the authors, without undue reservation.

## Ethics statement

The studies involving humans were approved by the Peking University Sixth Hospital. The studies were conducted in accordance with the local legislation and institutional requirements. The participants provided their written informed consent to participate in this study.

## Author contributions

N-nX: Conceptualization, Formal analysis, Funding acquisition, Writing – original draft, Writing – review & editing. T-tF: Conceptualization, Funding acquisition, Writing – original draft, Writing – review & editing. QL: Conceptualization, Supervision, Writing – review & editing. KF: Conceptualization, Methodology, Supervision, Writing – review & editing. RL: Formal analysis, Methodology, Supervision, Writing – review & editing. BS: Methodology, Supervision, Writing – review & editing. CW: Methodology, Supervision, Writing – review & editing. MM: Conceptualization, Methodology, Project administration, Resources, Supervision, Writing – review & editing.

## Footnotes

^1^https://www.h6world.cn
